# Overexpression of walldof transcription factor increases secondary wall deposition and alters carbon partitioning in poplar

**DOI:** 10.1186/1753-6561-5-S7-O35

**Published:** 2011-09-13

**Authors:** Isabel R Gerhardt, Silvia B Filippi, Vagner Okura, Jaime Coutinho, Ana P Rizzato, Karina Gui, Nathália Vessali, João HM Pontes, Thiago Cordeiro, Solange M Silva, Alexandre F Garcia, Paulo Arruda

**Affiliations:** 1 Canavialis Alellyx, Campinas, SP, Brazil, Present address: Embrapa Forestry, Colombo, PR, Brazil; 2 Canavialis Alellyx, Campinas, SP, Brazil; 3 Canavialis Alellyx, Campinas, SP, Brazil, Present address: State University of Campinas, Campinas, SP, Brazil

## Background

Of the total worldwide biomass produced by land plants, 70% is represented by cell walls [1]. In angiosperm trees, the fixed carbon is accumulated mainly in the secondary walls of xylem cells, which turns xylem cell wall formation a major carbon sink in trees[2]. The understanding of mechanisms that regulate carbon flux to the synthesis of cell wall components is a fundamental goal to maximize the amount of lignocellulosic biomass.

Several families of transcription factors have been shown to be important regulators of secondary wall biosynthesis, but how plants control and direct carbon partitioning among wall components still remains unclear [3,4].

In an attempt to identify transcription factors that participate in the regulation of carbon flux into secondary wall formation, a systematic search for poplar transcription factors expressed in a wood-preferred manner was performed. We have used the collection of over 400,000 ESTs from *Populus sp*. available at GenBank to determine the tissue-specific pattern of genes encoding transcription factors. Among these, a DOF (DNA-binding with one finger) domain transcription factor family member (which we named *WALLDOF*) that presents a cambium-preferred expression profiling was selected for further characterization.

DOF proteins are plant-specific transcription factors suggested to participate in the regulation of biological processes such as light-regulated gene expression, photosynthetic carbon assimilation, accumulation of seed-storage proteins, germination, response to phytohormones, guard cell-specific gene expression, flavonoid metabolism, and lipid biosynthesis [5,6].

In this work, we report that overexpression of *WALLDOF* in transgenic poplar is capable of increasing secondary wall deposition and altering carbon partitioning in poplar stems.

## Methods

The collection of over 400,000 ESTs from *Populus sp*. available in GenBank was searched for the tissue-specific pattern of expressed genes. For this purpose, ESTs from 100 cDNA libraries were grouped into 15 representative tissues/organs including apical shoot, bark, cambial zone, catkins, cultured cells, flower buds, leaves, mixed tissues, petioles, roots, seeds, shoot meristem, shoots, vegetative buds and xylem (including xylem and wood libraries). A set of clusters generated by CAP3 was searched for those composed of at least 60% of EST reads from libraries representing poplar cambial zone and stem tissues.

One cluster representing a DOF transcription factor family member with a cambium-preferred expression profiling was identified. Using a cDNA synthesized from total RNA from stems of *Populus deltoides* plants, the full-size ORF of the *WALLDOF* gene was cloned under the control of a 1.0 kb fragment of a *Populus deltoides* cinnamate 4-hydroxylase (C4H) promoter. Wild-type aspen hybrid (*Populus tremula x Populus alba*) was transformed with *Agrobacterium tumefaciens* carrying the construct.

## Results and conclusions

We obtained 12 transgenic poplar lines that were transferred to soil and grown in the greenhouse. After two months, they were harvested and screened for changes in lignin deposition by phloroglucinol staining of transverse stem sections. The initial screen showed that the intensity of the staining was higher in seven of twelve lines, suggesting a stronger lignification in those plants. Based on the initial lignin screen, four of the seven transgenic lines were randomly chosen for in-depth characterization. Overexpression of *WALLDOF* in transgenic poplar leads to a dramatic thickening of secondary wall and a reduction in the lumen area, which resulted in an increase of as much as 78% of the area occupied by wall in fiber cell cross-sections. Wall thickening at the expense of lumen area also resulted in higher wood density in transgenic lines. Moreover, *WALLDOF* overexpressing plants presented increased cellulose content, a general reduction in hemicellulose carbohydrates, mainly mannose and arabinose, and alteration in lignin composition. Starch content in stems was also reduced in transgenic plants.

Aiming to figure out which genes could be related to the altered phenotype, we proceed with a xylem transcriptome analysis of *WALLDOF* events using Affymetrix Genechip Poplar Genome Array. We have identified 825 genes that are differentially expressed, including a set of genes that participate in carbon and nitrogen metabolism.

The results indicate that *WALLDOF* is involved in carbon flux regulation and carbon partitioning through cell wall formation, and can contribute for increasing biomass production.
